# Traumatic transfemoral bowel evisceration following bull horn injury: a case report and review of literature

**DOI:** 10.1093/jscr/rjag901

**Published:** 2026-10-18

**Authors:** Venkata Avinash Kumar Vegi, Meraj Ahmed, Afzal Anees

**Affiliations:** Department of General Surgery, Jawaharlal Nehru Medical College and Hospital, Aligarh Muslim University, Aligarh202002, Uttar Pradesh, India; Department of General Surgery, Jawaharlal Nehru Medical College and Hospital, Aligarh Muslim University, Aligarh202002, Uttar Pradesh, India; Department of General Surgery, Jawaharlal Nehru Medical College and Hospital, Aligarh Muslim University, Aligarh202002, Uttar Pradesh, India

**Keywords:** bull horn injury, bowel evisceration, inguinal disruption, mesenteric tear, penetrating trauma

## Abstract

Bull horn injuries produce complex shearing and penetrating forces capable of causing occult abdominal wall disruption with visceral herniation. We report a rare case of bowel evisceration through the thigh following bull horn injury associated with pelvic fractures and traumatic inguino-femoral disruption. A 55-year-old female presented to the emergency with bowel loops protruding through a lacerated wound over the anterolateral aspect of the right thigh following a bull horn injury. Contrast-enhanced computed tomography of the abdomen and pelvis revealed herniation of ileal loops with mesentery through a large right lower abdominal wall defect with possible femoral canal involvement. Exploratory laparotomy revealed mesenteric tears and traumatic disruption of the inguinal region allowing bowel migration into the thigh. Reduction of bowel loops, repair of mesentery, peritoneal lavage and inguinal ligament repair were performed successfully. This case highlights the importance of suspecting occult abdominal wall and inguino-femoral disruption in atypical bowel evisceration patterns following animal-inflicted trauma.

## Introduction

Bull horn injuries are commonly encountered in rural regions [1] and are known to produce complex patterns of blunt and penetrating trauma resulting from tangential shearing, crushing, and penetrating forces [2]. Most reported injuries involve the abdomen, perineum, groin, and extremities [2, 3].

Traumatic abdominal wall hernias are uncommon [4], and bowel migration into the thigh through the femoral or inguinal region has only rarely been described in the literature [1]. Such injuries pose diagnostic and therapeutic challenges because associated mesenteric tears, occult bowel perforations, vascular compromise, and pelvic fractures may coexist [5, 6].

We present a case of traumatic transfemoral bowel evisceration through the thigh after bull horn injury, successfully managed by emergency exploratory laparotomy and repair.

## Case presentation

A 55-year-old woman presented to the emergency department following a bull horn injury to upper thigh. She complained of severe pain and protrusion of bowel loops through the right thigh. The patient had no other significant medical or surgical history.

At presentation, the patient was conscious, oriented, and hemodynamically stable.

The patient had undergone primary survey, was adequately resuscitated, and received primary antimicrobial (ceftriaxone) and tetanus prophylaxis. Local examination (Fig. 1) revealed eviscerated bowel loops protruding through a lacerated wound measuring ~13 × 5 cm over the anterolateral aspect of the right thigh, located nearly 10 cm inferior to the anterior superior iliac spine. Approximately 0.6 meters of bowel loops were visualized externally. The exposed bowel appeared edematous and viable.

**Figure 1:**
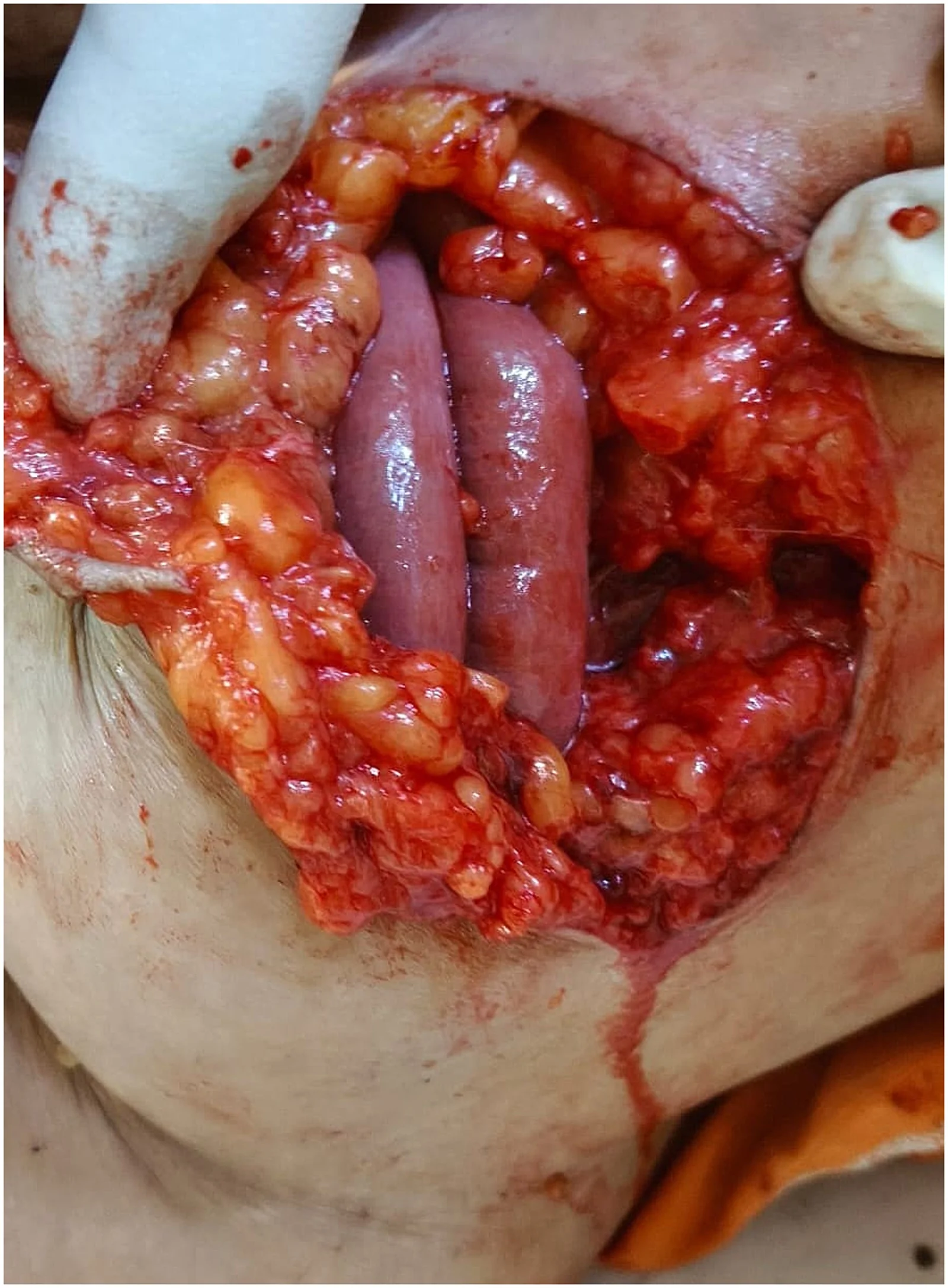
Image depicting laceration over the thigh with evisceration of bowel loops through it.

The patient had an estimated Injury Severity Score (ISS) of 26 and New Injury Severity Score (NISS) of 34, consistent with major polytrauma and classified as a Woods type 2 traumatic abdominal wall hernia.

Single-pass contrast-enhanced computed tomography (CT) of the abdomen and pelvis trauma protocol with multiplanar reconstruction (Fig. 2) demonstrated herniation of bowel loops along with mesentery through a defect measuring ~9.7 × 7.3 cm located between the rectus abdominis and lateral abdominal wall musculature extending into the right thigh region with fracture of the right ala of sacrum, anterior right acetabulum involving the superior pubic ramus and inferior pubic ramus.

**Figure 2:**
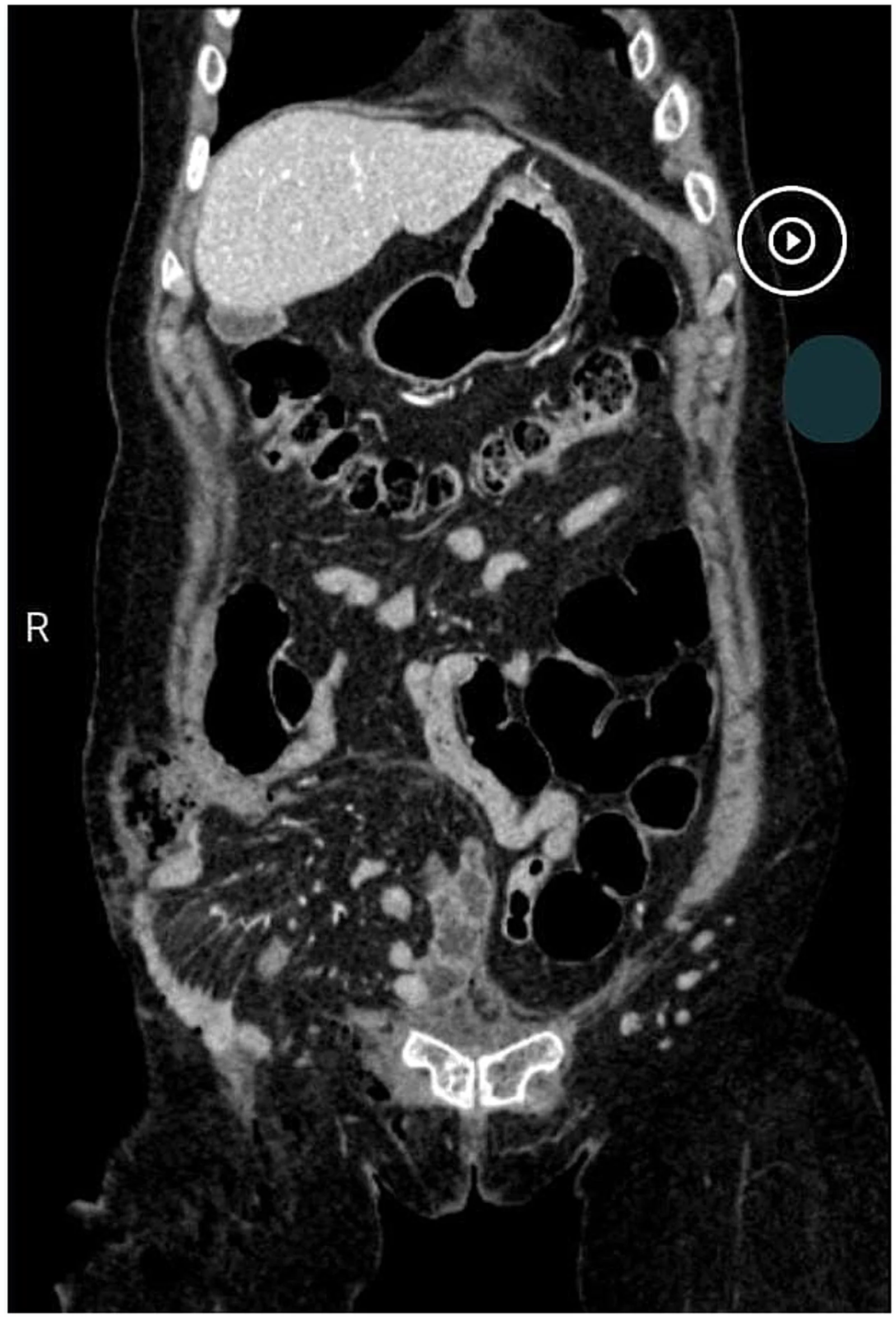
CT coronal section image depicting bowel evisceration.

A pelvic binder was applied and patient was taken to the operating room. Exploratory laparotomy revealed 60 cm of edematous and viable ileum eviscerated through the inguinal area with a tear in the inguinal ligament and a defect size of 10 × 8 cm; a 13 × 5 cm laceration over the right thigh 10 cm below the anterior superior iliac spine, three mesenteric defects of size 3 × 1, 3 × 1, and 5 × 1 cm (Fig. 3). We successfully performed reduction of bowel loops, repair of mesenteric defects with Vicryl 2–0 RB suture, peritoneal lavage, inguinal ligament repaired with Prolene 2–0 RB suture and primary closure of the laceration with placement of a closed suction drain. Mesh was not placed due to possible risk of contamination.

**Figure 3:**
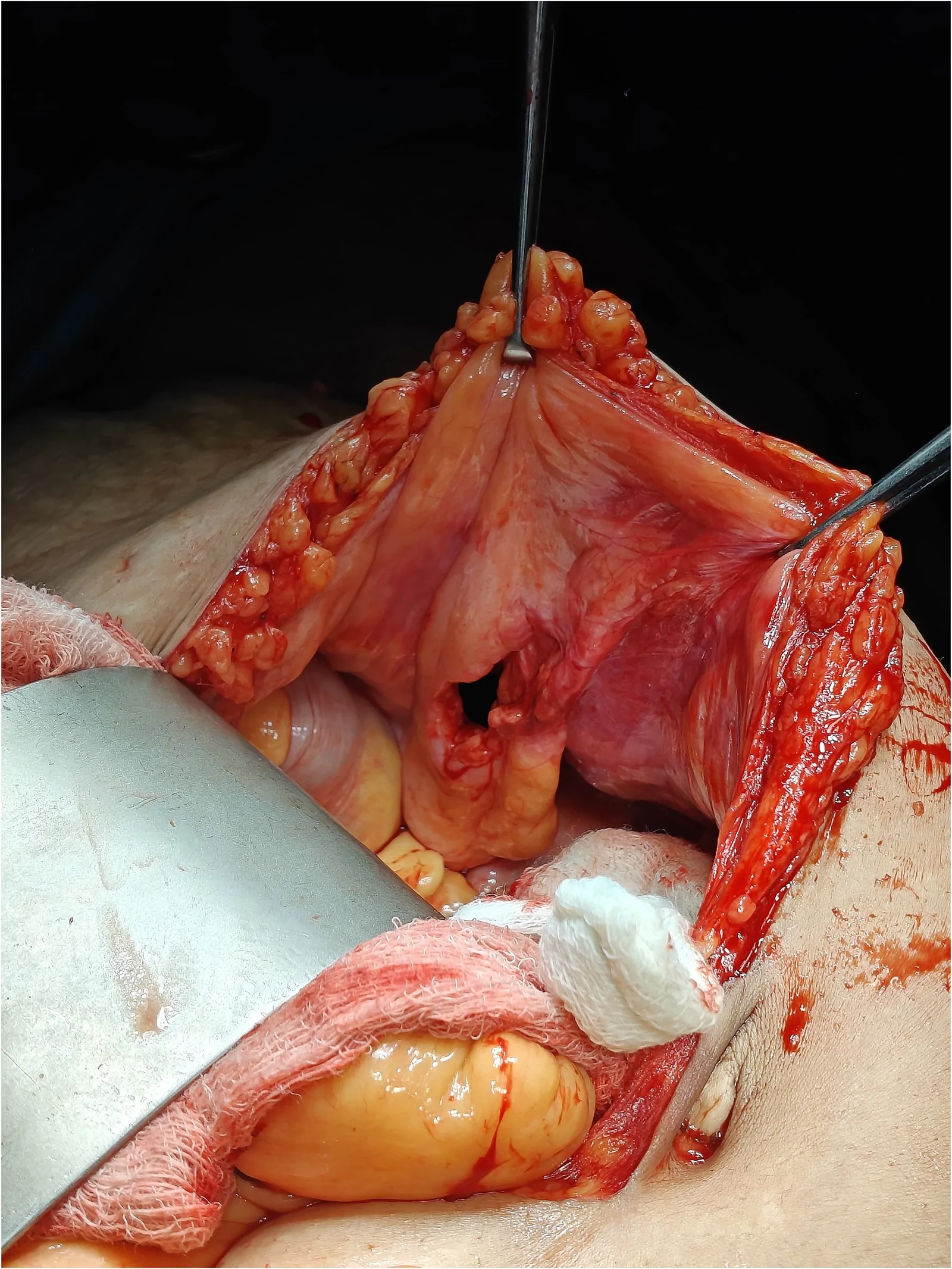
Intraoperative image depicting repair of inguinal ligament and defect at inguinal region.

The postoperative period was uneventful and the patient was discharged successfully on postoperative day 7. She presented to our OPD after 1 week for followup, she had no complaints and midline sutures were removed.

## Discussion

Bullhorn injuries are characterized by a unique mechanism involving penetration combined with tangential shearing forces [2]. These forces may create extensive underlying tissue disruption disproportionate to the size of the external wound [3]. The abdomen, groin, perineum, and thighs are frequently affected due to the height and manner of the animal’s charge [3].

Traumatic abdominal wall hernia is defined as herniation through disrupted musculature and fascia [7]. However, in the present case, there was overt soft tissue disruption with evisceration extending through the inguinal region into the thigh, making the injury pattern particularly unusual. Associated pelvic fractures may have contributed to further destabilization of the pelvic and inguinal anatomy.

In a retrospective analysis of 572 cases spanning four decades, Martínez Hernández *et al*. [8] characterized bull horn injuries as high-energy events that frequently result in trauma to the abdomen, perineum, and lower extremities.

In a 10-year retrospective review of 68 bullfighters identified from a cohort of 750, Rudloff *et al*. [9] demonstrated that bullfighting poses a substantial risk of severe, life-threatening injuries, particularly penetrating inguinal and perineal trauma involving the femoral vessels.

Sheldon *et al*. [10] analysed 287 incidents in the United States, reporting that bull-related encounters remain a significant cause of severe trauma and mortality, carrying a comparatively higher risk than injuries involving cows or tractor operations. An important differential consideration in this case is traumatic disruption of a pre-existing occult femoral or obturator hernia. However, absence of prior symptoms and the extensive acute traumatic fascial disruption support a diagnosis of traumatic inguino-femoral herniation rather than isolated pre-existing hernia.

CT played a crucial role in identifying associated mesenteric and bowel injuries [11]. The presence of extraluminal air foci mandated urgent surgical exploration to exclude occult perforation [12]. In our patient, timely laparotomy enabled reduction of viable bowel and repair of mesenteric defects before ischemic compromise occurred.

## Conclusion

Traumatic transfemoral bowel evisceration secondary to bull-horn injury constitutes an exceedingly rare surgical emergency arising from complex abdominal wall and inguino-femoral disruption. Optimal clinical outcomes are contingent upon a high index of clinical suspicion, rapid diagnostic imaging, and prompt surgical intervention. This case highlights the uncommon pathways of visceral herniation that may occur following high-energy animal-inflicted trauma and underscores the importance of meticulous evaluation for associated mesenteric and pelvic injuries.
